# Healthcare workers’ clinical knowledge on maternal and newborn care in Ethiopia: findings from 2016 national EmONC assessment

**DOI:** 10.1186/s12913-019-4758-x

**Published:** 2019-11-29

**Authors:** Theodros Getachew Zemedu, Aster Teshome, Yared Tadesse, Abebe Bekele, Emily Keyes, Patricia Bailey, Ana Lorena Ruano

**Affiliations:** 1 Health System and Reproductive Health Research Directorate, Ethiopian Public Health Institute, Addis Ababa, Ethiopia; 2 College of Medicine and Health Sciences, Institute of Public Health, University of Gondar, Gondar, Ethiopia; 3 Maternal and Child Health Directorate, Ministry of Health, Addis Ababa, Ethiopia; 4 Averting Maternal Death and Disability, Columbia University, NY and FHI 360, Columbia, North Carolina USA

**Keywords:** Knowledge, Maternal, Newborn, Ethiopia

## Abstract

**Background:**

Improving maternal and newborn health indicators are key if Ethiopia is to achieve the Sustainable Development Goals. To do so, women need access to skilled attendance at birth and emergency obstetric and newborn care. To maximize their impact, understanding gaps in workers’ knowledge is required to remedy the weakness. This assessment determines knowledge levels of clinical management of maternal and newborn healthcare and factors that influence knowledge.

**Methods:**

This study used data from the National Emergency Obstetric and Neonatal Care assessment conducted in 2016. Provider knowledge for MNH was assessed by interviewing providers. Respondents were scored on each question by calculating the number of correct responses provided out of the total possible answers, and standardizing this to a scale of 100. Mixed linear regression was used to determine individual and contextual factors associated with the score.

**Results:**

A total of 3800 interviews with complete data were included in this study. Most respondents were diploma midwives (73%), BSc midwives (11%) and diploma nurses (10%). On average, midwives scored 60 out of 100 on the question regarding the primary aspects of focused antenatal care and elements of a birth plan. Half of the midwives and health officers, and one-third of nurses knew to provide a loading dose of magnesium sulphate. Midwives scored 90% on the steps of active management of third stage of labor. In the mixed linear regression, working in a private for profit facility, health center/clinic, rural area, or in a facility with a protocol on referral/counter referral predicted lower knowledge scores. More positive scores were associated with work environments that had a computer, internet, and protocols on safe abortion care, management of selected obstetric topics, integrated management of pregnancy, childbirth, postnatal, and newborn, care for low birth weight including kangaroo mother care, and treatment of infection in young infants.

**Conclusion:**

With regard to most knowledge related questions, health officers and midwives scored similarly. Providers scored substantially better on routine intrapartum and newborn care than on aspects related to care for complications. A substantial proportion of providers indicated that they would never give a loading dose of magnesium sulphate.

## Background

Although there has been substantial progress in reducing maternal and neonatal mortality in the last 20 years, it is estimated that about 287,000 women and 2.6 million newborns die of pregnancy and birth-related complications each year [1]. About 85% of the global burden of maternal and neonatal deaths happen in low and middle-income countries (LMICs), with a disproportionate share of them taking place in sub-Saharan Africa [2]. Ethiopia is no exception, and country’s estimated maternal mortality ratio (MMR) is 676 deaths per 100,000 live births, and the neonatal mortality rate (NMR) is 37 deaths per 1000 live births [3]. Among the main causes of maternal death are postpartum hemorrhage, hypertensive disorders of pregnancy, infections, and obstructed labor. For newborns, they are prematurity, intrapartum complications and infections [4]. Many of these deaths could be averted through access to skilled institutional care.

Improving maternal and newborn health indicators are key if Ethiopia is to meet the Sustainable Development Goals (SDGs). To achieve this, the country needs to ensure access to skilled attendance at birth and emergency obstetric and newborn care (EmONC) for all women and infants who need it. EmONC interventions are a cost-effective way to reduce maternal and neonatal morbidity and mortality in poor resource settings [5], and basic EmONC (BEmONC) alone can avert 40% of intrapartum related neonatal deaths, as well as a significant proportion of maternal mortality [6]. According to the health sector transformation plan, (HSTP) Ethiopia has set a target of reducing the country’s MMR to 199/100,000 and the NMR to 12/1000 by 2020 [4]. To attain these goals, the country has intensified EmONC capacity building trainings, which have been implemented since 1998. The goal of these training sessions, which targets nurses, midwives and health officers, is to address the knowledge and skill gaps that providers face during complicated deliveries.

Despite the favorable impact on maternal and newborn survival that EmONC interventions have, current understanding of healthcare worker knowledge remains limited throughout the region. In Mali and Kenya, primary healthcare workers reported little knowledge or competency of EmONC, particularly when it comes to hypertensive patients, postpartum infections and obstetric emergencies in general [7–9]. Insufficient knowledge regarding the diagnoses of emergency conditions, like postpartum hemorrhage (PPH) and birth asphyxia, was higher among nurses and midwives from Addis Ababa [5]. However, these studies focused on specific cadres of healthcare workers and were carried out in urban settings or based on small samples. Currently, we have limited information on knowledge among all healthcare cadres in all regions of Ethiopia. Therefore, the purpose of this paper is to evaluate gaps related to clinical knowledge on routine maternal and newborn care, treatment of complications and the factors that influence knowledge.

## Methods

### Study setting

We used cross-sectional data from the National Emergency Obstetric and Neonatal Care assessment, a collaboration between the Averting Maternal Mortality and Disability program (AMDD) and the Ethiopian Institute of Public Health (EPHI). Data were collected between the months of May and October of 2016 in all 9 regions and in the 2 city administrations of the country.

Questionnaires were paper-based, followed by performing data entry later in the day in the field. This process was called CAFE (computer-assisted field editing) and each team had one tablet. Face-to-face interviewing was used to collect the data. The data were sent to the Central Office using the Internet File Streaming System (IFSS). These files were extracted, reviewed, and checked for errors and inconsistencies. Secondary editing was done by two data managers located at EPHI and if errors or inconsistencies were found, teams were asked to correct and resend the data.

### Participants

In total, the survey included 3804 health facilities. All eligible public hospitals (referral, general, primary) and health centers, and all eligible for-profit and not-for-profit private facilities (hospitals, Maternal and Child Health (MCH), specialty centers, MCH specialty clinics, and higher clinics) were included in the study. Eligibility was determined by three characteristics: 1) health facilities classified as higher clinic or above as per the regulation set by the Food, Medicine and Health Care Administration and Control Authority of Ethiopia (FMHACA); and 2) the facility reported having attended births in the last 12 months; and 3) the facility was deemed functional, i.e. it was not under construction and was at least minimally operational. The target population for this study consisted of all health worker cadres. The target population for this study consisted of a single functioning birth attendant per facility. Three selection criteria based on practicality were applied: the individual who had attended the largest number of deliveries in the last month, regardless of health worker cadre, and who was physically present and consented during the data collection team visit to the facility. If no births had been reported in the facility in the previous 30 days, the last 60 days were considered. Thus, the pool of birth attendants consisted largely of mid-level professionals since doctors and specialists are generally not found in health centers and health centers were far more numerous than hospitals. The data collection teams were health workers trained for two week on the data collection tools. Data collection occurred from May to November 2016.

### Measurement

Based on the set of knowledge questions administered to healthcare workers, extracted from the national clinical guidelines for Emergency Obstetric Care that covered a range of maternal and newborn topics, such as routine and emergency care during pregnancy, intrapartum care, essential newborn care, and care for sick newborns, a knowledge level score was generated. Average summary scores were then calculated for each specific question included in the topic area. Each knowledge question had multiple “correct” answers; that is, answers that respondents were expected to provide spontaneously. If a correct answer was not offered spontaneously, the interviewer coded the response as “not mentioned.” If a spontaneous answer did not appear as one of the pre-coded options, it was not taken into consideration for scoring purposes. Respondents were scored on each question by calculating the number of correct responses mentioned out of the total possible, and standardizing this to a scale of 100. Antenatal care, for example, was represented by three questions, the first of which asked respondents to list the components of focused antenatal care that had eight possible responses. In order to handle confounding effects, a mixed-effect linear regression model was used to determine individual and contextual factors associated with the knowledge score. The regression coefficient tells us how much the summary knowledge score is expected to increase when the independent variable increases by one, holding all other independent variables constant. In addition to the univariate model, we fitted three models. Model I was fitted on facility level factors, Model II on provider-level factors, and Model III included both facility and provider level factors. Akaike’s information criterion (AIC) and Bayesian information criterion (BIC) were run to measure the models fit and complexity. For the given models fitted on the same data, the model with the smallest value of the information criterion is considered to be the best.

## Results

### Socio demographic characteristics of health care providers

From the possible maximum total of 3804 healthcare providers, 3800 interviews with complete data were included in this study, for a response rate of 99.9%. Most respondents were diploma midwives (73%), followed by BSc midwives (11%), and diploma nurses (10%). Most respondents were female (63%), though the sex distribution varied by cadre, facility type and managing authority. The average respondent was 26 years old, had been posted to the current facility for 2 years, and had been practicing with current qualification for just over 3 years. Midwives reported attending around 21 deliveries per month, exceeded by 4 medical doctors who reported delivering 24 deliveries per month. The monthly average of deliveries conducted by nurses was between 11 and 12, followed by health officers who attended on average 9 deliveries per month (Table 1).

**Table 1:** Percent distribution of providers according to health worker cadre, facility type, and managing authority, and distribution of providers according to demographic and professional characteristics, ^1^ Ethiopia

	Providers interviewed		Characteristics			Professional experience		
			Sex		Mean age (in years)	Mean number of deliveries attended in past month	Mean number of years at current facility	Mean number of years since receiving professional qualification
	n	%	Female	Male				
National	3800	100	63	37	25.7	19.3	2.0	3.2
Health worker cadre								
MD (general practitioner)	4	0	50	50	36.3	24.5	1.8	9.7
Midwife (BSc)	407	11	37	63	25.8	21.1	1.4	2.4
Midwife (Diploma)	2786	73	74	26	25.5	20.8	1.9	3.2
Nurse (BSc)	86	2	42	58	28.1	12.1	3.2	3.7
Nurse (Diploma)	370	10	35	65	26.7	11.1	2.8	4.6
Health officer	143	4	25	76	25.8	8.8	1.5	2.0
Other^2^	4	0	0	100	32.3	1.5	1.0	3.8
Facility type								
Referral/specialized hospitals	30	1	50	50	27.8	25.8	3.0	4.8
General hospitals	103	3	68	32	28.4	16.2	3.0	5.1
Primary hospitals	160	4	48	52	26.7	18.7	1.8	3.3
MCH specialty centers	23	1	87	13	31.0	10.2	2.9	6.8
Health centers	3455	91	64	36	25.5	19.4	1.9	3.1
MCH specialty clinics	16	0	69	31	28.6	7.5	1.2	4.7
Higher clinics	13	0	69	31	28.3	8.6	3.8	5.5
Managing authority								
Public/government	3658	96	63	37	25.6	19.6	1.9	3.1
Private-for-profit	83	2	82	18	28.5	6.4	3.2	6.7
Private-not-for-profit^3^	59	2	66	34	29.6	12.8	3.5	5.5

^1^Non-response varies and does not exceed 3.6. Non-responses are excluded

^2^Other health worker cadres include emergency surgical officers and health extension practitioners

^3^Includes NGO, faith-based, or mission facilities

### Knowledge of care during pregnancy, intrapartum, abortion and violence, and newborn care

Knowledge score were, aggregated by cadre, and presented in Table 2. Due to small number of respondents for medical doctors and other health professional category, results were omitted from this table.

**Table 2:** Provider knowledge scores (out of 100) related to a set of questions on antenatal care, intrapartum, newborn, postnatal, postpartum, abortion care and violence by health worker cadre, Ethiopia

	Midwives (*n* = 3193)	Nurses (*n* = 456)	Health officers (*n* = 143)	Total
ANC				
Primary aspects of focused antenatal care (*n* = 8)	58	50	57	55
Elements of a birth plan (*n* = 5)	59	46	53	53
Women who require a special care plan (*n* = 10)	32	30	42	35
Intrapartum				
ROUTINE CARE				
Admission and referral requirement before onset of labor (*n* = 6)	42	37	48	42
Observations to monitor labor progress (n = 10)	75	56	70	67
Steps of active management of the third stage of labor (AMTSL) (n = 3)	90	68	83	80
CARE FOR COMPLICATIONS				
Management principles for women with ≥1 previous caesarean delivery and uterine scar (n = 3)	49	42	57	49
Management principles for women with PROM (n = 5)	38	26	47	37
Management principles for women with heavy bleeding after delivery (n = 10)	52	40	50	47
Loading dose of magnesium sulphate* (n = 4)	52	33	51	45
Newborn				
ROUTINE CARE				
Immediate newborn care (*n* = 13)	68	60	66	65
Key counselling messages for cord care (n = 4)	51	44	49	48
Timing of first bath (n = 1)	97	93	94	95
CARE FOR COMPLICATIONS				
Care for low birth weight newborns (< 2000 g) (*n* = 9)	38	32	39	36
Signs and symptoms of newborn infection and sepsis (n = 5)	42	36	54	44
Signs of critical illness for a newborn requiring referral (n = 10)	32	31	40	34
Diagnosis of birth asphyxia (n = 4)	46	40	51	46
Steps of neonatal resuscitation (n = 8)	51	39	46	45
Postnatal and postpartum				
Checks for the baby at a postnatal visit (*n* = 8)	49	44	54	49
Checks for the mother at a postpartum visit (*n* = 12)	40	32	41	38
Abortion and violence				
Immediate complications of unsafe abortion (n = 5)	51	47	60	53
Steps to treat woman with complications of an unsafe or incomplete abortion (n = 10)	46	40	52	46
Information to provide to clients treated for an unsafe or incomplete abortion (*n* = 7)	42	37	46	42
Steps to take when treating a woman who is a victim of rape (n = 8)	35	33	42	37
Total knowledge score (out of 100)	52	43	54	50

Provider knowledge score for when (under what circumstances) to give a loading dose of magnesium sulphate is not measured among providers who said they would never give the drug (12% of midwives, 20% of nurses, 13% of health officers)

Knowledge score on 4 medical doctors and 4 other health professionals were not presented in this table.

Health workers scored above 50 on two out of the three antenatal questions.

Health workers tended to score higher on questions related to routine intrapartum or newborn care than they did on care for maternal or newborn complications. Scores were highest for questions regarding the timing of a newborn’s first bath (score of 95) and the steps of Active Management of Third Stage of Labour (AMTSL) (score of 80) and lowest for which women require a special care plan (score of 35) or care for low birth weight newborns (score of 36). Nurses consistently scored lower than health officers and midwives (Table 2).

**Fig. 1:**
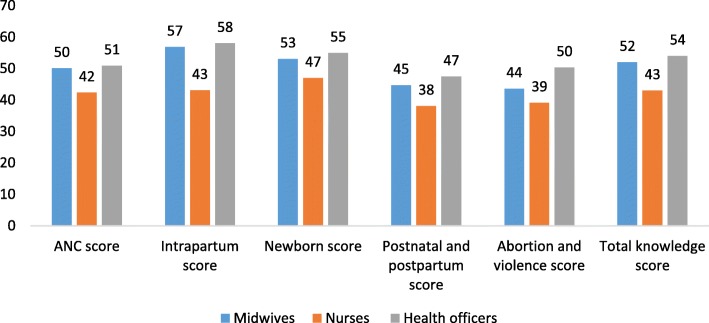
Health providers’ summary knowledge score on topics of maternal and newborn health care, Ethiopia

The overall knowledge score for maternal and newborn health care among midwives, nurses, and health officers were 52, 43, and 54 respectively. In general, midwives and health officers consistently scored higher than nurses in all topic areas of maternal and newborn health care (Fig. 1).

### Determinants of health providers’ knowledge on maternal and newborn care

The determinants of health providers’ knowledge on maternal and newborn care are presented in Table 3. Model I has the smallest AIC and BIC (Table 3), therefore Model I based on facility level factors had the best fit and was the least complex.

**Table 3:** Multivariable mixed-effects linear regression analysis to identify the provider and facility-level determinants of clinical knowledge on maternal and newborn care in Ethiopia (*n* = 3800)

Variables	Univariate model		Model I		Model II		Model III	
	β Coef.	*P*-value	β Coef.	*P*-value	β Coef.	*P*-value	β Coef.	*P*-value
*Provider-level variables*								
Providers cadre								
Medical Doctors	1				1		1	
Health Officers	0.050	0.576			0.090	0.299	0.082	0.340
Midwife BSC and Above	0.084	0.344			0.131	0.125	0.109	0.203
Midwife diploma	0.016	0.857			0.073	0.389	0.061	0.470
Nurse BSC	0.026	0.771			0.097	0.262	0.079	0.364
Nurse diploma	−0.070	0.433			0.002	0.978	−0.007	0.937
Total experience	−0.001	0.011			0.000	0.132	0.000	0.167
NO of facilities posted previously	0.000	0.179			0.000	0.769	0.000	0.785
Experience in current facility	0.000	0.907			0.000	0.958	0.000	0.946
Providers age	0.001	0.192			0.000	0.543	0.000	0.874
No. of delivery attended in last month	0.000	0.004			0.000	0.627	0.000	0.794
Providers sex								
Female	1				1		1	1
Male	0.018	< 0.001			0.028	**< 0.0001**	0.029	**< 0.0001**
No. of maternal and newborn services delivered in last 3 month	0.011	< 0.001			0.003	**< 0.0001**	0.006	**< 0.0001**
No. of training received on maternal and newborn services	0.003	< 0.001			0.254	**< 0.0001**	0.003	**< 0.0001**
*Facility-level variables*								
Managing authority								
Public	1.000		1.000				1.000	1.000
Private for profit	0.002	0.892	− 0.049	**< 0.001**			−0.049	**0.005**
Private for non-profit	0.006	0.707	−0.014	0.419			−0.012	0.495
Types of facility								
Hospital/MCH specialty center	1.000		1.000				1.000	1.000
Health center/clinics	−0.064	< 0.0001	− 0.043	**< 0.0001**			−0.023	0.054
Facility location								
Urban	1		1				1	1
Rural	−0.037		−0.019	**< 0.0001**			−0.021	**0.001**
Availability of computer								
No	1		1				1	1
Yes	0.031	< 0.0001	0.010	**0.041**			−0.001	0.917
Availability of internet								
No	1		1				1	1
Yes	0.058	< 0.0001	0.020	**0.043**			0.016	0.164
Availability of safe abortion care guideline								
No	1		1				1	1
Yes	0.037	< 0.0001	0.015	**0.002**			−0.005	0.400
Availability of protocol on management of selected obstetric topics								
No	1		1				1	1
Yes	0.031	< 0.0001	0.012	**0.014**			0.002	0.723
Availability of protocol on integrated management of pregnancy, childbirth, postnatal, and newborn								
No	1		1				1	1
Yes	0.029	< 0.0001	0.011	**0.030**			−0.002	0.797
Availability of protocol on care for LBW including KMC								
No	1		1				1	1
Yes	0.027	< 0.0001	0.012	**0.017**			0.015	**0.018**
Availability of protocol on treatment of infection in young infants (IMNCI)								
No	1		1				1	1
Yes	0.019	< 0.0001	0.005	0.343			−0.002	0.724
Availability of protocol on referral and counter referral								
No	1		1				1	1
Yes	0.011	0.028	−0.012	**0.018**			−0.006	0.340
*Random effect*								
Variance (SE)			0.016 (0.0004)		0.014 (0.0004)		0.014 (0.0004)	
Model Fitness								
Log Likelihood			2428.184		1469.503		1483.823	
AIC			− 4828.368		− 2907.007		− 2911.647	
BIC			− 4740.981		− 2816.673		− 2753.589	

Model I was fitted on facility level variables, Model II on provider-level variables, and Model III included both facility and provider level variables

Bold entries have signficant values at 5%

Negative coefficients, indicating a lowering of the knowledge score, were observed when providers worked in a private for-profit facility, in a health center/clinic, in a rural facility, and where there was a protocol on referral and counter referral. On the other hand, positive coefficients were found when providers worked in facilities with a computer, with internet, with protocols on safe abortion care, on management of selected obstetric topics, on integrated management of pregnancy, childbirth, postnatal, and newborn, and on care for Low Birth Weight (LBW) infants including Kangaroo Mother Care (KMC) (Table 3).

## Discussion

In this study we aimed to assess the level of health worker knowledge regarding essential standard components of maternal and newborn care. Health officers and midwives scored similarly and higher than nurses on most knowledge questions. Substantial proportion of providers who indicated that they would never give a loading dose of magnesium sulphate. Only average scores were observed regarding health workers’ knowledge on routine newborn care. All provider cadres scored substantially better on aspects of routine intrapartum and newborn care than on aspects of care for intrapartum or newborn complications, which might be expected given that the vast majority of the health workers were mid-level practitioners. Nevertheless, all were front-line providers who are faced with maternal and newborn complications, if only to stabilize and refer them to doctors and specialists posted at hospitals. Knowledge levels were higher among providers posted in hospitals, suggesting they might have greater exposure to women and newborns with complications and to more highly skilled staff.

Magnesium sulphate was expected to be provided in all type of cadres under this study, however, there was a substantial proportion of providers who indicated that they would never give a loading dose of magnesium sulphate; among nurses, this was one in five respondents. Magnesium sulphate has been on the World Health Organization’s (WHO) essential medicines list since 1996, and it is an affordable drug [10]. However, magnesium sulphate has not achieved widespread usage in developing countries. This is due to lack of public awareness of the drug, lack of adequate service-provider training, and not all facilities had magnesium sulphate in stock [11, 12].

Only average scores were observed regarding health workers’ knowledge on routine newborn care. This means that women in the postnatal period may not receive adequate information on immediate newborn care, hygienic cord care, timing of first bath for the newborn, and care for newborns with low birth weight.

A study undertaken in Mali to determine individual and contextual factors associated with emergency obstetric and neonatal care services suggests that the existence of clinical guidelines or protocols is an important factor associated with knowledge of healthcare providers [7]. Even if they used small sample size compared with us, similarly, the present study showed a significant positive association between the existence of guidelines in the workplace and knowledge. It could be that healthcare professionals actually read and know the guidelines better when they have them at hand. In contrast with the current study, female gender revealed a significant positive association with provider knowledge in Rwanda where the focuse were the final-year medical students at university suggesting female students had a higher likelihood of demonstrating retention and competency compared with their male counterparts [13]. In a study on knowledge of birth preparedness and complication readiness among doctors, nurses and midwives in Benin, awareness and training on birth preparedness and educational status were significant predictors of knowledge [14]. This study used small sample size compared to our study which is around four hundred health care providers.

The national reach and the large number of health providers included in the study were important strengths of this study. But it was not without some limitations. The assessment was based on respondents’ reports, rather than direct observation, which might have led to some reporting error. Most of the questions had multiple correct answers that required spontaneous responses; this may have biased scores towards the lower end because of respondents’ fatigue (due to interview or lack of sleep if on night duty), or the interviewer was in a hurry and failed to adequately probe and encourage the provider to think of other responses. If, for example, all questions had been posed as multiple choice answers, the scores might have been higher.

Steps were taken to ensure the quality of the data collected. All data collectors received the same two-week training. Regular supervision of questionnaire completion, along with using electronic data collection approaches contributed to high quality. In addition, the electronic transfer system we utilized regularly reviewed the quality of the data and provided feedback while the data collectors were still in the field.

Our findings suggest that in-service and refresher trainings and dissemination of tailored clinical guidelines for the management of maternal and newborn care are vital to update health workers’ knowledge levels and self-confidence in their skills, which may lead to them serving the community better. Since the workers in hospitals had higher scores than workers from health centers, it is recommended that healthcare workers do rotations in hospitals; also, it would be important to ensure access to internet and to clinical guidelines in all facilities.

Although midwives made up the bulk of the sample of respondents, where there are no midwives and nurses or health officers are expected to attend births, we see that nurses were the least knowledgeable. This is both an argument to ensure midwives at every health center and that nurses get more specialized obstetric training. The study compared knowledge scores among midwives, nurses and health officers who have different pre-service training backgrounds. However, during practice all health workers are expected to offer standard care in the provision of maternal and newborn services.

This study have a number of policy implications. Clinical decision making involves combining the knowledge arising from one’s clinical expertise that would be improved by provision of guidelines, patient preferences, and research evidence within the context of available resources including computer and internet.

## Conclusion

Health officers and midwives scored similarly on most knowledge questions. All provider cadres scored substantially better on aspects related to routine intrapartum and newborn care than on aspects of complicated care. There was a substantial proportion of providers who indicated that they would never give a loading dose of magnesium sulphate; a better understanding of why nurses and others had this response would lead to better care for both the woman and her baby.

In a mixed linear regression, working in a private for profit facility, in health centers/clinics, in rural facilities or in a facility with referral and counter referral guidelines predicted lower knowledge score, while the availability of a computer, the internet, and the existence of protocols, were found to be independent positive predictors of providers’ knowledge on maternal and newborn care.

## Data Availability

The data for this manuscript was provided by the Ethiopian Public Health Institute (EPHI). The primary data is possible to access after getting ethical approval from the EPHI institutional review board. Interested researchers may contact the Director of the Health System and Reproductive Health Research Directorate at the EPHI, Addis Ababa, Ethiopia through email; abebe1277belay@gmail.com .

## Abbreviations

AIC: Akaike Information Criterion
AMDD: Averting Maternal Mortality and Disability program
AMTSL: Active Management of Third Stage of Labour
BIC: Bayesian Information Criterion
EPHI: Ethiopian Institute of Public Health
FMHACA: Food, Medicine and Health Care Administration and Control Authority of Ethiopia
HSTP: Health Sector Transformation Plan
IMNCI: Integrated Management of Newborns and Child Infections
KMC: Kangaroo Mother Care
LBW: Low Birth Weight
LMICs: Low and Middle-Income Countries
MCH: Maternal and Child Health
MMR: Maternal Mortality Rate
NMR: Neonatal Mortality Rate
PPH: Postpartum Hemorrhage
SDG: Sustainable Development Goal
WHO: World Health Organization
